# Self-esteem and self-compassion status of migraine patients in Turkey: a multi-center study by Turkish Headache Study Group

**DOI:** 10.3389/fneur.2025.1566423

**Published:** 2025-07-25

**Authors:** Basak Karakurum-Goksel, Ruhsen Ocal, Mert Van, Ozlem Coskun, Cihad Karaaslan, Serap Ucler, Figen Gokcay, Nese Celebisoy, Hadiye Sirin, Aysin Kisabay-Ak, Aysegul Seyma Saritas, Tuba Cerrahoglu-Sirin, Buse Rahime Hasirci-Bayir, Esme Ekizoglu, Elif Kocasoy-Orhan, Derya Bayram, Nermin Tanik, Sebnem Bicakci, Vesile Ozturk, Levent Ertugrul Inan, Kubra Mehel Metin, Yasemin Eren, Babur Dora, Emel Oguz-Akarsu, Necdet Karli, Emel Ur-Ozcelik, Arife Cimen Atalar, Rabia Gokcen Gozubatik-Celik, Belgin Mutluay, Elif Ilgaz-Aydınlar, Pinar Yalinay Dikmen, Sencer Semercioglu, Ufuk Emre, Osman Cagin Buldukoglu, Busra Er, Bekir Burak Kilboz, Seray Ibis, Sibgetullah Yagiz, Huzeyfe Koklu, Ibrahim Kamaci, Gulshan Aliyeva, Basak Elcin Ates, Muge Mercan Kara, Fatma Zehra Altunc, Ilgin Kaya, Cagla Sisman, Secil Ozkan

**Affiliations:** ^1^Baskent University, Faculty of Medicine, Turgut Noyan Adana Hospital Department of Neurology, Adana, Türkiye; ^2^Department of Neurology, Antalya Training and Research Hospital, University of Health Sciences, Antalya, Türkiye; ^3^Gazi University, Faculty of Medicine, Department of Neurology, Ankara, Türkiye; ^4^Kırıkkale University, Faculty of Mediciene, Department of Neurology, Kırıkkale, Türkiye; ^5^Prof. Dr. Cemil Tascioglu City Hospital, Department of Neurology, Istanbul, Türkiye; ^6^Ege University, Faculty of Medicine, Department of Neurology, Izmır, Türkiye; ^7^Celal Bayar University, Faculty of Medicine, Department of Neurology, Manisa, Türkiye; ^8^Sisli Hamidiye Etfal Training and Research Hospital, Department of Neurology, Istanbul, Türkiye; ^9^University of Health Sciences, Haydarpasa Numune Training and Research Hospital, Department of Neurology, Istanbul, Türkiye; ^10^Istanbul University, Faculty of Medicine, Department of Neurology, Istanbul, Türkiye; ^11^Adana City Training and Research Hospital, Department of Neurology, Adana, Türkiye; ^12^Yozgat Bozok University, Faculty of Medicine, Department of Neurology, Yozgat, Türkiye; ^13^Cukurova University, Faculty of Medicine, Department of Neurology, Adana, Türkiye; ^14^Dokuz Eylul University, Faculty of Medicine, Department of Neurology, Izmır, Türkiye; ^15^Ankara Training and Research Hospital, Department of Neurology, Ankara, Türkiye; ^16^Diskapi Training and Research Hospital, Department of Neurology, Ankara, Türkiye; ^17^Akdeniz University, Faculty of Medicine, Department of Neurology, Antalya, Türkiye; ^18^Uludag University, Faculty of Medicine, Department of Neurology, Bursa, Türkiye; ^19^Istanbul Kanuni Sultan Suleyman Training and Research Hospital, Department of Neurology, Istanbul, Türkiye; ^20^Istanbul Bakirkoy Prof. Dr. Osman Mental Health and Neurological Diseases Training and Research Hosoital, Clinic of Neurology and Neurosurgery, Istanbul, Türkiye; ^21^Acıbadem University, School of Medicine, Department of Neurology, Istanbul, Türkiye; ^22^Istanbul Training and Research Hospital, Department of Neurology, Istanbul, Türkiye; ^23^University of Health Sciences, Antalya Training and Research Hospital, Department of Gastroenterology, Antalya, Türkiye; ^24^Gazi University, Faculty of Medicine, Department of Public Health, Ankara, Türkiye

**Keywords:** migraine, self-esteem, self-compassion, depression, anxiety

## Abstract

**Introduction:**

Migraine, characterized by recurrent attacks, often necessitates a holistic approach for effective management. Treatment strategies aimed at enhancing self-esteem and self-compassion have been shown to reduce pain intensity in patients with chronic pain. This study aimed to investigate self-esteem and self-compassion in individuals with migraine.

**Materials and methods:**

This multicentre, cross-sectional, case–control study included migraine patients evaluated at headache-specialized outpatient clinics across 22 centers in different regions of Turkey. 901 migraine patients and 901 healthy, matched controls were included. Neurologists specializing in headache disorders administered the Rosenberg Self-Esteem Scale, Self-Compassion Scale, Beck Depression Inventory, and Beck Anxiety Inventory to all participants. Data were compiled at a central site and subjected to statistical analysis.

**Results:**

Migraine patients exhibited significantly lower self-esteem scores compared to healthy controls (*p* = 0.001 for both). Additionally, Beck Depression Inventory and Beck Anxiety Inventory scores were significantly higher in migraine patients (*p* = 0.001 for both). Although the mean self-compassion scores of migraine patients and healthy controls were comparable, a significant decline in self-compassion was observed among patients with higher migraine attack frequency.

**Discussion:**

In conclusion, our findings indicate that migraine patients exhibit significantly higher levels of anxiety and depression compared to healthy controls. Self-esteem scores were also found to be lower in the migraine group. Although self-compassion scores were similar between the two groups, a noteworthy decline in self-compassion was observed in patients with higher migraine attack frequency. These results suggest that increased migraine severity may negatively impact patients’ emotional resilience, highlighting the potential value of integrating psychological support into migraine management, particularly for those with frequent attacks.

## Introduction

Migraine, with an annual global incidence exceeding 1 million cases, is the second leading cause of disability across all age groups and the primary cause of disability in women aged 15–49 (1). Beyond its physical impact, migraine is frequently comorbid with psychiatric disorders, including depression, anxiety, bipolar disorder, post-traumatic stress disorder, and personality disorders, and is associated with a higher incidence of suicide attempts. Additionally, psychological factors such as anxiety, emotional distress, and uncertainty significantly contribute to the manifestation of migraine headaches (2). Diagnosis is established based on the International Classification of Headache Disorders-3 (ICHD-3) criteria (3).

Prior studies emphasize the role of self-compassion (SC), defined by kindness, mindfulness, and a sense of common humanity, in headache disorders. SC is associated with reduced stress, anxiety, depression, pain perception, and disability. The evaluation of SC in chronic pain disorders is a growing area of research, as lower SC levels have been linked to increased pain severity. Self-compassion therapy (SCT) has been shown to effectively reduce pain intensity in migraine patients while also improving emotional regulation (4, 5). Recognizing one’s experiences as part of the shared human condition and maintaining a balanced awareness of painful thoughts and emotions is a key component of self-compassion (6). Evidence further suggests a negative association between SC and symptoms of depression and anxiety (7–9). Moreover, self-compassion has been implicated in physiological processes related to pain regulation, including vagally mediated heart rate variability and the oxytocin-endorphin system. The assessment of SC in chronic diseases has gained increasing attention in recent years (9, 10).

Rosenberg defines self-esteem (SE) as an indicator of self-acceptance, personal satisfaction, and overall self-worth. It plays a crucial role in daily life, influencing an individual’s perseverance and resilience in the face of challenges. SE reflects a person’s positive or negative self-perception (11). Recent studies further highlight the relationship between positive self-esteem, physical literacy, and physical activity in college students (12).

Previous findings indicate a positive correlation between low self-compassion and chronic pain. While supportive and treatment-based strategies targeting SC have been found beneficial in managing chronic pain, current literature lacks data on the status of migraine patients in terms of SC and SE (5, 7).

This study aims to assess self-compassion and self-esteem in migraine patients and explore their impact on migraine severity and frequency. Evaluating these factors in migraine patients may provide valuable insights for guiding individuals toward cognitive behavioral therapy or psychological support.

## Materials and methods

The study was designed as a multicenter, prospective, case–control study. Ethics committee approval number 2022-179 was received for the study from Health Sciences University Antalya Training and Research Hospital. Before the study, a meeting was held with 22 centers from the Turkey Headache Study Group that agreed to participate in the study and a common data form was created for data entry. A voluntary consent form was obtained from the patients to participate in the study.

This multicenter, prospective, case–control study obtained ethical approval (2022-179) from the Health Sciences University Antalya Training and Research Hospital. Collaboration with 22 centers from the Turkey Headache Study Group facilitated the creation of a standardized data form for the study. Patient consent was acquired voluntarily. Demographic details and test responses were meticulously recorded. Data underwent thorough validation in a single center before entry into the SPSS system. Exclusion criteria involved psychiatric diagnoses or medication use (antidepressants, beta-blockers, antipsychotics) that could impact test results. Face-to-face interviews were conducted using the Rosenberg self-esteem scale, Self-compassion scale, Beck depression scale, and Beck anxiety scale, all previously validated in Turkish (13–16). Initially, 1,200 patients with migraine and 964 control participants were enrolled. After excluding individuals with incomplete data or those who did not meet the predefined exclusion criteria, 901 patients with migraine and 901 controls were included in the final analysis. “After data verification, the test results were entered into the SPSS software. Scores from the Rosenberg Self-Esteem Scale, the Self-Compassion Scale, the Beck Depression Inventory, and the Beck Anxiety Inventory were compared between 901 patients with migraine and 901 healthy control participants.

### Rosenberg Self-Esteem Scale

Utilizing a 4-point Likert scale, participants respond to 10 items ranging from strongly agree to strongly disagree. The scale serves as a self-report measure, assessing global self-esteem through statements on self-worth and self-acceptance. The total score is calculated, leading to the creation of three subgroups based on the score: normal and low self-esteem (11). On the Rosenberg Self-Esteem Scale, the total score ranges from 0 to 30, where higher scores reflect greater self-esteem. A score between 15 and 25 was interpreted as normal self-esteem, whereas a score below 15 was considered indicative of low self-esteem. The validity and reliability of the Turkish version of this scale have been previously established (11, 16).

### Self-Compassion Scale

The Self-Compassion Scale consists of 26 items encompassing six subscales: self-kindness, self-judgment, common humanity, isolation, mindfulness, and over-identification. The validity and reliability of the Turkish version of the scale have been previously confirmed. Participants rate each item using a five-point Likert scale (ranging from “never” to “always”), and total scores are compared with those of a healthy control group. Higher scores indicate greater levels of self-compassion (17, 18).

### Beck Depression Inventory

The Beck Depression Inventory (BDI) comprises 21 items, each presenting four response options. Participants are asked to select the statement that best reflects their mood over the past week, including the day of assessment. Each item is scored from 0 to 3, and the total score is obtained by summing all responses. Higher total scores reflect greater severity of depressive symptoms. Based on total scores, individuals are classified into four categories: normal, mild depression, moderate depression, and severe depression (14, 19).

### Beck Anxiety Inventory

The Beck Anxiety Inventory (BAI) is a 21-item self-report measure assessing the severity of anxiety symptoms experienced over the past week. Each item is rated on a 0–3 scale, with higher total scores indicating more severe anxiety. The scale classifies individuals into four categories: normal, mild, moderate, and severe anxiety levels. Standardized data entry procedures were applied across both the migraine and control groups, allowing for consistent and comprehensive comparisons across all psychological measures (13, 15).

### Statistical analysis

Statistical analyses were conducted using the Statistical Package for the Social Sciences (SPSS) for Windows, version 26.0 (IBM SPSS Inc., Chicago, IL, United States). The distributional characteristics of the variables were assessed for normality through both visual (histograms and probability plots) and analytical methods (Kolmogorov–Smirnov and Shapiro–Wilk tests).

Descriptive statistics for categorical variables were presented as frequencies and percentages. Continuous variables were evaluated for normality using both visual and analytical approaches as mentioned above. Variables exhibiting a normal distribution were expressed as mean ± standard deviation (mean ± SD), whereas non-normally distributed variables were presented as median along with minimum and maximum values (median [min–max]).

The Pearson chi-square test was employed for the analysis of categorical variables. For independent continuous variables not conforming to a normal distribution, the Mann–Whitney U test was applied. Spearman’s rank correlation coefficient was used to assess correlations between non-normally distributed continuous variables.

Correlation coefficients were interpreted as follows: 0.00–0.25 as weak, 0.26–0.50 as moderate, 0.51–0.75 as strong, and 0.76–1.00 as very strong. A *p*-value of less than 0.05 was considered statistically significant throughout the study.

## Results

Demographic data, along with responses to the Rosenberg Self-Esteem Scale, the Self-Compassion Scale, the Beck Depression Inventory, and the Beck Anxiety Inventory, were collected from a total of 1,928 participants (964 patients with migraine and 964 healthy controls). Data collection was conducted across 22 centers by neurologists specialized in headache disorders. Prior to completing the questionnaires, a face-to-face briefing session was held to standardize the study procedure. Upon completion of data collection, all forms were reviewed at a central coordination site. Participants with incomplete or inaccurate data were excluded from the final analysis.

After matching for age and sex, a total of 901 migraine patients and 901 healthy controls were included in the final comparison for the Rosenberg Self-Esteem Scale and the Self-Compassion Scale.

As shown in Table 1, there was no statistically significant difference in age or gender distribution between the migraine and control groups. Although migraine is more prevalent among females in the general population, the gender distribution in our sample was not significantly different between groups (*p* = 0.152).

**Table 1:** Demographic characteristics of the study groups.

Variable	Control group	Patient group	*p*-value
Age (Mean ± SD)	31.21 ± 8.02	31.41 ± 7.55	0.091
Age (Median, min–max)	29 (18–69)	31 (18–65)	—
Gender (Female %)	71.5%	74.5%	0.152

Mann–Whitney U and Chi-square tests were used as appropriate.

Among the 901 patients with migraine, aura status was reported by 884 individuals (98.1%). Of these, 13.6% (*n* = 120) experienced migraine with aura, while 86.4% (*n* = 764) reported migraine without aura. Aura status was missing in 17 participants (1.9%).

In this study, Rosenberg Self-Esteem Scale scores were compared between the migraine and healthy control groups. Analysis of the coding distribution in the patient group revealed that the proportion of individuals with low self-esteem was significantly higher compared to the control group. No statistically significant difference was found between the two groups in terms of self-compassion scores. However, individuals in the migraine group exhibited significantly higher levels of depressive and anxiety symptoms compared to the control group (Table 2). Analysis of the Beck Depression Inventory (BDI) scores revealed that the proportion of participants with moderate-to-severe depressive symptoms was significantly higher in the migraine group (33.3%) compared to the control group (16.6%) (*p* < 0.001). Similarly, Beck Anxiety Inventory (BAI) results showed that 37.6% of individuals in the migraine group reported moderate-to-severe anxiety symptoms, which was significantly greater than the 17.6% observed in the control group (*p* < 0.001).

**Table 2:** Comparison of psychological scale scores between groups.

Scale	Control group	Patient group	*p*-value
Low self-esteem (RSES, % low)	7.8%	15.4%	<0.001
Self-compassion (RSCS, median)	72 (24–111)	72 (16–119)	0.377
Depression (BDI, moderate-to-severe %)	16.6%	33.3%	<0.001
Anxiety (BAI, moderate-to-severe %)	17.6%	37.6%	<0.001

These findings indicate that individuals with migraine experience considerably higher levels of both depressive and anxiety symptoms compared to healthy controls.

When examining the relationship between monthly migraine attack frequency and self-compassion scores, it was found that individuals experiencing more frequent attacks (>15 per month) had significantly lower self-compassion scores (Table 3).

**Table 3:** Self-compassion scores by attack frequency.

Attack group	*N*	Mean	Median	Min–max	SD	*p*-value
<15 Attacks per month	716	71.20	73.0	16–105	12.37	
≥15 Attacks per month	148	68.36	68.5	26–119	12.95	0.002

*p-value calculated using Mann–Whitney U test.*

## Discussion

This study represents a pioneering investigation into the relationship between self-esteem (SE) and self-compassion (SC) in adult migraine patients in Turkey. While previous studies have explored the effectiveness of Self-Compassion Therapy (SCT) and mindfulness in adolescent migraine patients, research focused on adult populations, specifically examining SE and SC, remains limited (20–23).

Our study also highlights the association between migraine and mental health conditions such as depression and anxiety. Both the Beck Depression Inventory (BDI) and Beck Anxiety Inventory (BAI) scores of the patient group were statistically significantly higher than those of the control group. These findings are consistent with existing literature (24–28), which suggests that anxiety disorders are more prevalent in migraine patients than depression. A recent systematic review reported that anxiety disorders are four times more common in individuals with migraine compared to those without. This data supports the findings of our study, emphasizing the need for careful evaluation of anxiety in migraine patients, given its relatively high co-occurrence (26).

The relationship between SE and migraine has been investigated primarily in adolescents by Ucar et al. (20), revealing that both migraine and tension-type headache patients exhibit low self-esteem. However, to our knowledge, there is no existing literature on the relationship between SE, SC, and migraine in adult populations. This study may be beneficial in this regard. In adolescent migraine patients, SE was found to be lower compared to healthy peers, impacting emotional, physical, social, and familial domains. Additionally, low self-esteem (SE) was linked to more intense headache symptoms and other psychological disorders (20, 27, 29). In our study, 15.0% (*n* = 144) of migraine patients and 7.7% (*n* = 74) of the control group had low SE, indicating a statistically significant difference. These findings suggest that adult migraine patients share similar characteristics to adolescents in terms of SE. Given that low SE is an amendable condition through cognitive-behavioral therapy (CBT) and psychiatric support, we recommend that all migraine patients be assessed for SE, with those exhibiting low SE referred for further psychiatric evaluation.

The relationship between SC and chronic pain is an area of ongoing research, yet a lack of standardization necessitates further studies. SC is believed to influence the affective states of patients, and interventions aimed at improving SC have shown efficacy in both SC and pain management among chronic pain patients. Studies on SC in migraine patients are scarce. In an investigation of 168 migraine patients conducted by Vasigh and colleagues at a single center, no significant relationship was found between self-compassion (SC) and pain. However, the small sample size was a notable limitation of this research (21).

Another study by Barcakh et al. (24) found that CBT reduced pain levels in migraine patients, and mindfulness techniques have been shown to improve SC (25). Our study, which included 901 migraine patients and control groups across multiple centers, demonstrated a statistically significant correlation between SC and migraine.

Although the overall levels of self-compassion (SC) in migraine patients were comparable to those of the control group, a significant decline in SC scores was observed in patients with a higher frequency of migraine attacks. This finding suggests that self-compassion may be preserved in general among migraine patients but becomes compromised in those experiencing more frequent episodes. One possible explanation is that recurrent, unpredictable pain episodes may lead to increased self-criticism, frustration, and emotional exhaustion, which are known to inversely affect self-compassion. Previous studies in chronic pain populations have similarly reported reduced SC in individuals with persistent or severe symptoms. Therefore, frequent migraine attacks may serve as a stressor that gradually erodes patients’ ability to respond to themselves with kindness and understanding. These findings highlight the potential value of interventions such as mindfulness-based therapy or self-compassion training, particularly for patients with chronic and frequent migraine.

The present study demonstrates that as the frequency of migraine attacks increases, self-compassion scores tend to decline. This finding suggests that effective preventive management of migraine attacks may help preserve patients’ self-compassion levels. Maintaining higher self-compassion could contribute positively not only to pain perception but also to the overall psychological well-being of migraine patients. Therefore, integrating strategies that target both headache frequency and psychological resilience may enhance treatment outcomes.

The multi-center, prospective design of this study, combined with its novel investigation into SE and SC in migraine patients, represents its key strengths.

Despite the strengths of this study, including a large and demographically matched sample of 901 migraine patients and 901 healthy controls, several limitations should be acknowledged. First, the cross-sectional design of the study precludes the establishment of causal relationships between migraine severity and psychological variables such as self-compassion, self-esteem, anxiety, and depression. Second, although data were collected from multiple centers, the assessment of migraine attack severity was based on self-reported data, which may be subject to recall bias. Finally, although validated psychological scales were used, the study did not include clinical psychiatric evaluations, which might have provided more comprehensive insights into comorbid mental health disorders.

## Conclusion

Migraine patients may experience low self-esteem and self-compassion. Recognizing these factors can expand the range of therapeutic options available to healthcare providers treating migraine patients. In addition to low SE and SC, depression and anxiety frequently coexist with migraine, compounding the burden of this already debilitating condition. The coexistence of low SE, low SC, depression, and anxiety underscores the importance of evaluating each migraine patient for these conditions and addressing them in the management of migraine.

## Data Availability

The raw data supporting the conclusions of this article will be made available by the authors, without undue reservation.

## References

[1] Zhang N Robbins MS. Migraine. Ann Intern Med. (2023) 176:ITC4–ITC16. doi: 10.7326/AITC202301170 36623287

[2] Minen MT Begasse De Dhaem O Kroon Van Diest A Powers S Schwedt TJ Lipton R. Migraine and its psychiatric comorbidities. J Neurol Neurosurg Psychiatry. (2016) 87:741–9. doi: 10.1136/jnnp-2015-312233, PMID: 26733600

[3] IHS. Headache Classification Committee of the International Headache Society (IHS). The international classification of headache disorders, 3rd edition. Cephalalgia. (2018) 38:1–211. doi: 10.1177/0333102417738202 29368949

[4] Wren AA Somers TJ Wright MA Goetz MC Leary MR Fras AM. Self-compassion in patients with persistent musculoskeletal pain: relationship of self-compassion to adjustment to persistent pain. J Pain Symptom Manag. (2012) 43:759–70. doi: 10.1016/j.jpainsymman.2011.04.014, PMID: 22071165

[5] Lanzaro C Carvalho SA Lapa TA Valentim A Gago B. A systematic review of self-compassion in chronic pain: from correlation to efficacy. Span J Psychol. (2021) 24:24–6. doi: 10.1017/SJP.2021.22, PMID: 33840398

[6] Neff K. Self-compassion: an alternative conceptualization of a healthy attitude toward oneself. Self Identity. (2003) 2:85–101. doi: 10.1080/15298860309032

[7] MacBeth A Gumley A. Exploring compassion: a meta-analysis of the association between self-compassion and psychopathology. Clin Psychol Rev. (2012) 32:545–55. doi: 10.1016/j.cpr.2012.06.003 22796446

[8] Biber DD Ellis R. The effect of self-compassion on the self-regulation of health behaviors: a systematic review. J Health Psychol. (2019) 24:2060–71. doi: 10.1177/1359105317713361, PMID: 28810473

[9] Rockliff H Gilbert P Mcewan K Lightman S Glover D. A pilot exploration of heart rate variability and salivary cortisol responses to compassion-focused imagery. Clin Neuropsychiatry. (2008) 5:132–9.

[10] Rockliff H Karl A Mcewan K Gilbert J Matos M Gilbert P. Effects of intranasal oxytocin on ‘compassion focused imagery’. Emotion. (2011) 11:1388–96. doi: 10.1037/a0023861, PMID: 21707149

[11] Rosenberg M Schooler C Schoenbach C Rosenberg F. Global self-esteem and specific self-esteem: different concepts, different outcomes. Am Sociol Rev. (1995) 60:141–56. doi: 10.2307/2096350

[12] She X Gao TY Ma RS Tang D Zhong H Dong HL. Relationship among positive self-esteem, physical literacy, and physical activity in college students: a study of a mediation model. Front Psychol. (2023) 17:1097335. doi: 10.3389/fpsyg.2023.1097335 PMC10230059 37265948

[13] Ulusoy M Sahin N Erkman H. Turkish version of the Beck anxiety inventory: psychometric properties. J Cogn Psychother. (1998) 12:28–35.

[14] Tukus L.. Turkish Validation of *The Self Esteem Rating Scale-Short Form* (Tipta Uzmanlik Tezi). Kocaeli University, Faculty of Medicine, Kocaeli. (2010). Available online at: https://tez.yok.gov.tr/ (Accessed March 15, 2024).

[15] Neff KD. The development and validation of a scale to measure self-compassion. Self Identity. (2003) 2:223–50. doi: 10.1080/15298860309027, PMID: 26979311

[16] Deniz M Kesici S Sumer AS. The validity and reliability of the Turkish version of the self-compassion scale. Soc Behav Pers. (2008) 36:1151–60. doi: 10.2224/sbp.2008.36.9.1151

[17] Beck AT Ward CH Mendelson M Mock J Erbaugh J. An inventory for measuring depression. Arch Gen Psychiatry. (1961) 4:561–71. doi: 10.1001/archpsyc.1961.01710120031004, PMID: 13688369

[18] Beck AT Epstein N Brown G Steer RA. An inventory for measuring clinical anxiety. Psychometric properties. J Consult Clin Psychol. (1988) 56:893–7. doi: 10.1037//0022-006x.56.6.893, PMID: 3204199

[19] Hisli N. Beck depresyon envanterinin üniversite öğrencileri için geçerliliği ve güvenilirliği. Psikoloji Dergisi. (1989) 7:3–13.

[20] Ucar HN Tekin E Tekin U. Pain severity and psychosocial quality of life in adolescents with migraine and tension-type headache: mediation by perceived expressed emotion and self-esteem. J Oral Facial Pain Headache. (2021) 35:62–71. doi: 10.11607/ofph.2768, PMID: 33730128

[21] Vasigh A Tarioman A Soltani B Borji M. The relationship between mindfulness and self-compassion with perceived pain in migraine patients in Ilam. Arch Neurosci. (2019) 6:e91623. doi: 10.5812/ans.91623

[22] Kemper KJ Heyer G Pakalnis A Binkley PF. What factors contribute to headache-related disability in teens? Pediatr Neurol. (2016) 56:48–54. doi: 10.1016/j.pediatrneurol.2015.10.024, PMID: 26810775 PMC5248515

[23] Chapin HL Darnall BD Seppala EM Doty JR Hah JM Mackey SC. Pilot study of a compassion meditation intervention in chronic pain. J Compassionate Health Care. (2014) 1:4. doi: 10.1186/s40639-014-0004-x, PMID: 27499883 PMC4972045

[24] Barchakh Z Mardani Valandani Z Khorvash F. The effectiveness of compassion-focused therapy for improving emotional control and reducing the severity of pain in migraine patients. Pract Clin Psychol. (2021) 9:51–60. doi: 10.32598/jpcp.9.1.740.1

[25] Estave PM Margol C Beeghly S Anderson R Shakir M Coffield A. Mechanisms of mindfulness in patients with migraine: results of a qualitative study. Headache. (2023) 63:390–409. doi: 10.1111/head.14481, PMID: 36853655 PMC10088163

[26] Duan S Ren Z Xia H Wang Z Zheng T Li G. Associations between anxiety, depression with migraine, and migraine-related burdens. Front Neurol. (2023) 14:1090878. doi: 10.3389/fneur.2023.1090878, PMID: 37181566 PMC10166814

[27] George A Minen MT. Episodic migraine and psychiatric comorbidity: a narrative review of the literatür. Curr Pain Headache Rep. (2023) 27:461–9. doi: 10.1007/s11916-023-01123-4 37382869

[28] Irimia P Garrido-Cumbrera M Santos-Lasaosa S Aguirre-Vazquez M Correa-Fernández J Colomina I. Impact of monthly headache days on anxiety, depression and disability in migraine patients: results from the Spanish atlas. Sci Rep. (2021) 11:8286. doi: 10.1038/s41598-021-87352-2, PMID: 33859216 PMC8050317

[29] Esposito M Gallai B Parisi L Castaldo L Marotta R Lavano SM. Self-concept evaluation and migraine without aura in childhood. Neuropsychiatr Dis Treat. (2013) 9:1061–6. doi: 10.2147/NDT.S49364, PMID: 23950647 PMC3742352

